# Supplementary material for: NAK-associated protein 1/NAP1 activates TBK1 to ensure accurate mitosis and cytokinesis
Source: J Cell Biol. 2023 Dec 7;223(2):e202303082. doi: 10.1083/jcb.202303082 (PMC10702366; doi:10.1083/jcb.202303082)

Figure 1D

Scramble control and TANK KD HeLa

Lane order for the blot:
Scramble ShRNA TANK KD ShRNA
Async. Mitotic Async. Mitotic

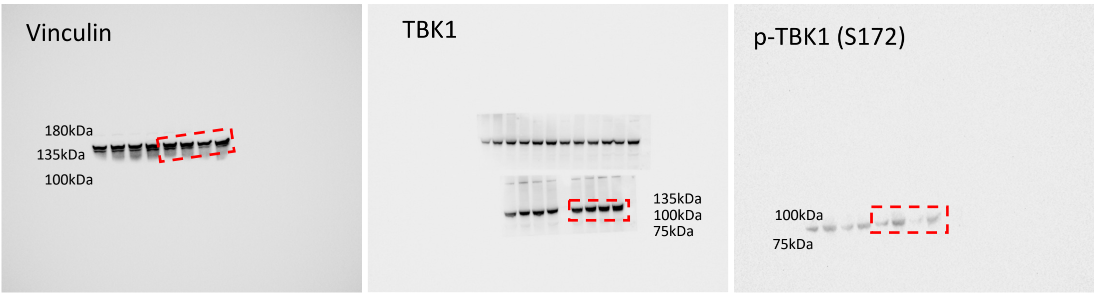

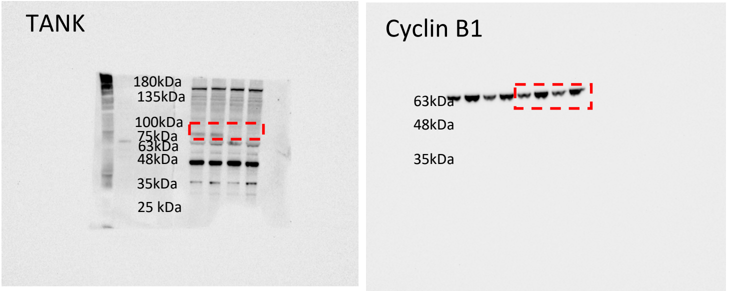

Figure 1F

Scramble control and SINTBAD KD HeLa

Lane order for the blot:
Scramble ShRNA SINTBAD ShRNA
Async. Mitotic Async. Mitotic

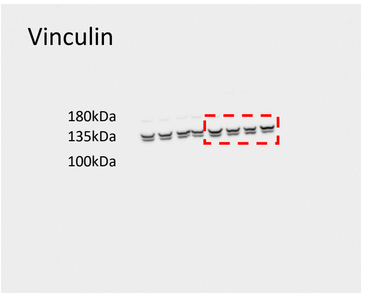

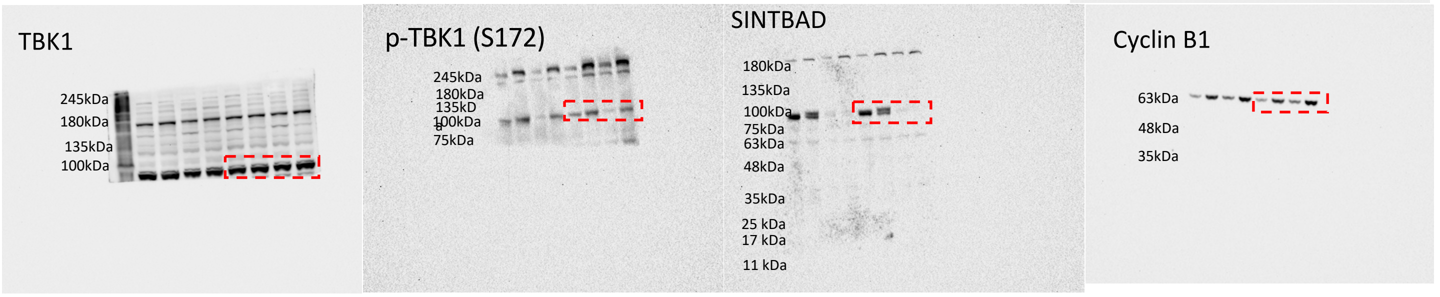

Figure 1I

Scramble control and NAP1 KD HeLa

Lane order for the blot:
Scramble ShRNA NAP1 ShRNA
Async. Mitotic Async. Mitotic

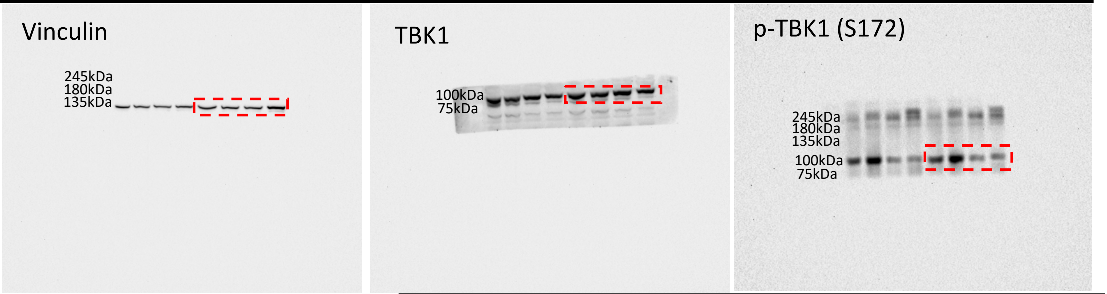

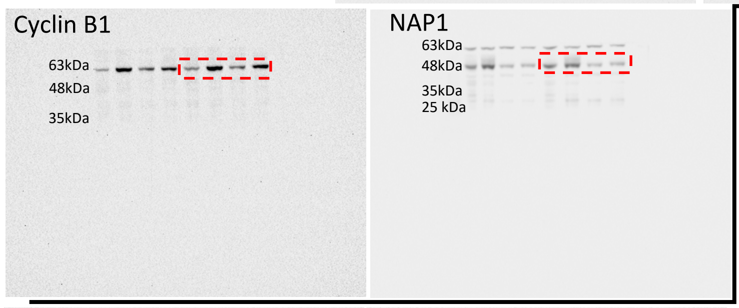

Figure 1K

WT HeLa and Penta KO HeLa

Lane order for the blot:
WT HeLa Penta KO HeLa
Async. Mitotic Async. Mitotic

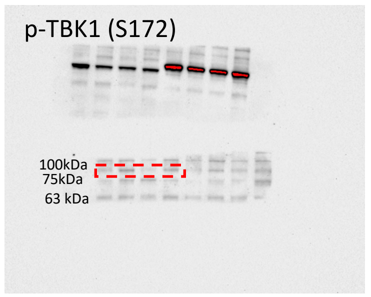

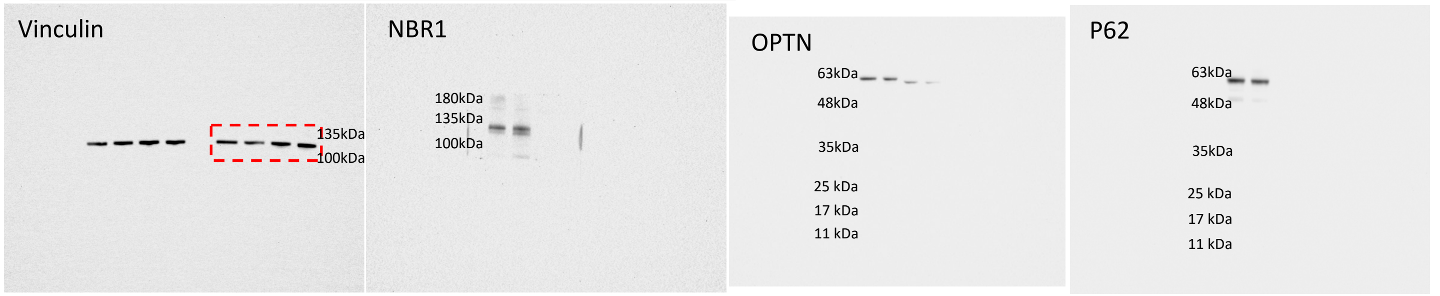

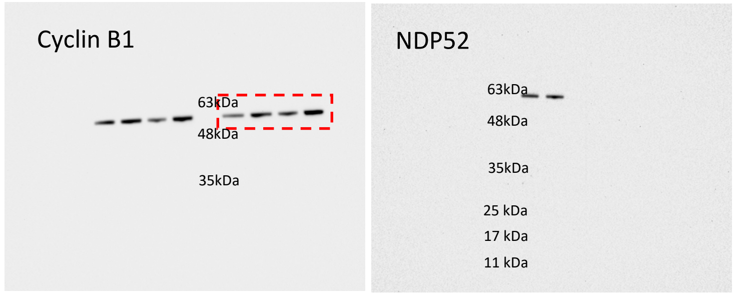

Supplement: SourceData F1 — is the source file for Fig. 1. [file JCB_202303082_SourceDataF1.pdf]
