# Supplementary material for: NAK-associated protein 1/NAP1 activates TBK1 to ensure accurate mitosis and cytokinesis
Source: J Cell Biol. 2023 Dec 7;223(2):e202303082. doi: 10.1083/jcb.202303082 (PMC10702366; doi:10.1083/jcb.202303082)

Figure 2A

WT and NAP1 KO HeLa

Lane order for the blot:

WT HeLa		NAP1 KO #10		NAP1 KO #12	
Async.	Mitotic	Async.	Mitotic	Async.	Mitotic

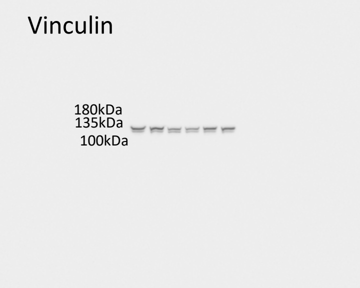

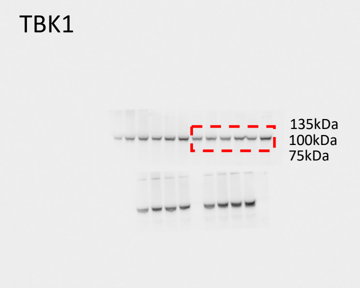

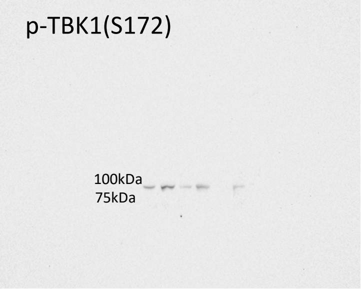

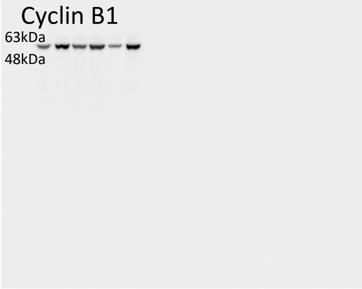

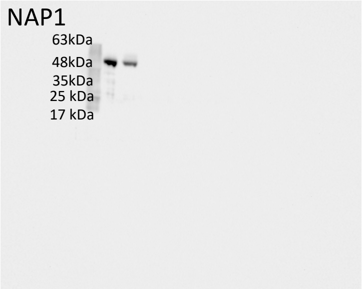

Figure 2C

WT and NAP1 KO and NAP1 Rescue HeLa

Lane order for the blot:

WT HeLa		NAP1 KO #12		NAP1 KO rescue	
Async.	Mitotic	Async.	Mitotic	Async.	Mitotic

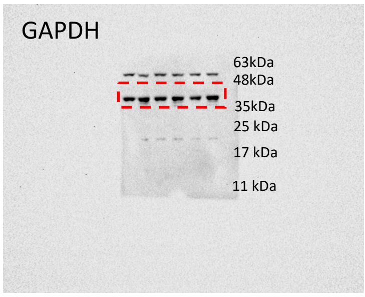

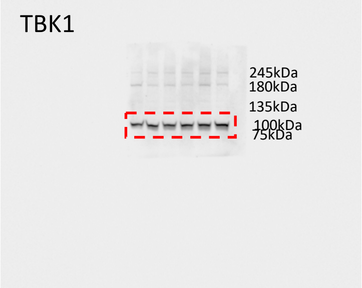

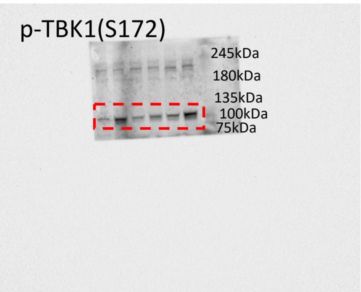

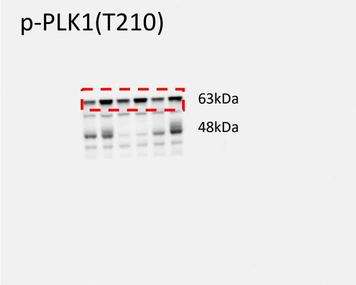

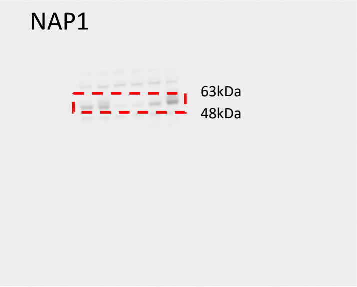

Supplement: SourceData F2 — is the source file for Fig. 2. [file JCB_202303082_SourceDataF2.pdf]
