# Supplementary material for: NAK-associated protein 1/NAP1 activates TBK1 to ensure accurate mitosis and cytokinesis
Source: J Cell Biol. 2023 Dec 7;223(2):e202303082. doi: 10.1083/jcb.202303082 (PMC10702366; doi:10.1083/jcb.202303082)

Figure 3B

UT and dTAG T DLD-1

Lane order for the blot:

<u>UT</u> <u>dTAG T</u>

Async. Mitotic Async. Mitotic

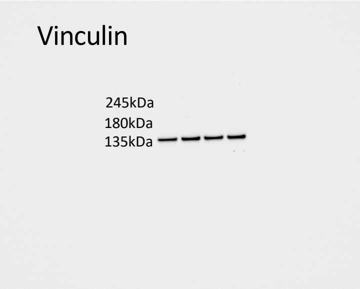

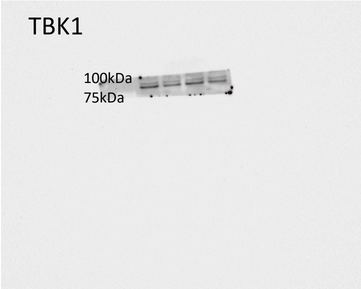

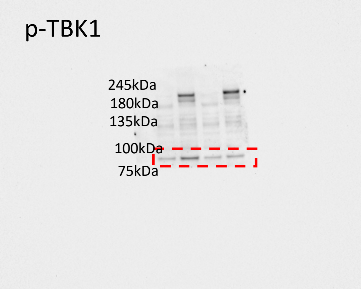

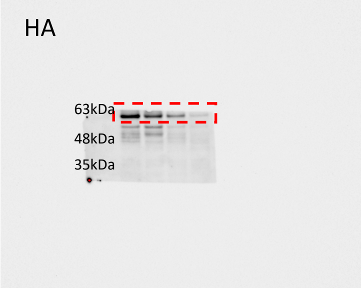

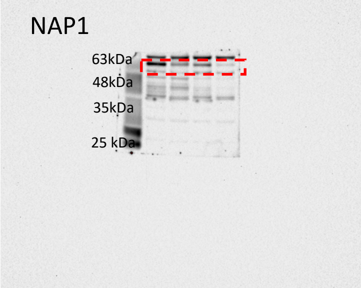

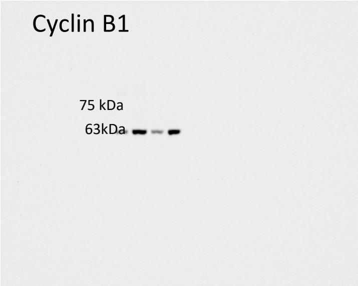

Figure 3E

dTAG NAP1 20 hours treatment

Lane order for the blot:

<u>dTAG NAP1 DLD-1</u>

UT 20hrs T

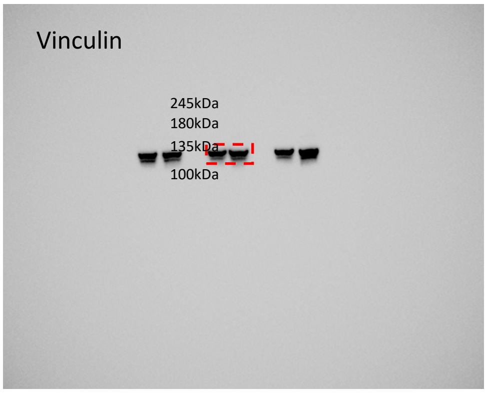

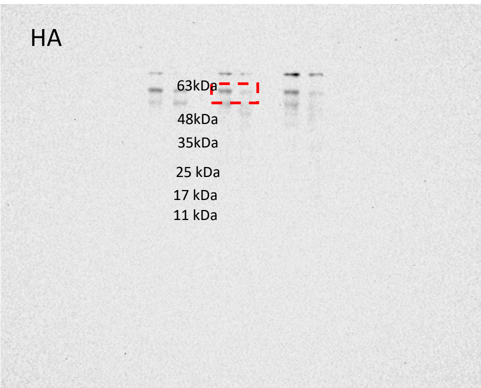

Supplement: SourceData F3 — is the source file for Fig. 3. [file JCB_202303082_SourceDataF3.pdf]
