# Supplementary material for: NAK-associated protein 1/NAP1 activates TBK1 to ensure accurate mitosis and cytokinesis
Source: J Cell Biol. 2023 Dec 7;223(2):e202303082. doi: 10.1083/jcb.202303082 (PMC10702366; doi:10.1083/jcb.202303082)
Supplement: SourceData F4 — is the source file for Fig. 4. [file JCB_202303082_SourceDataF4.pdf]

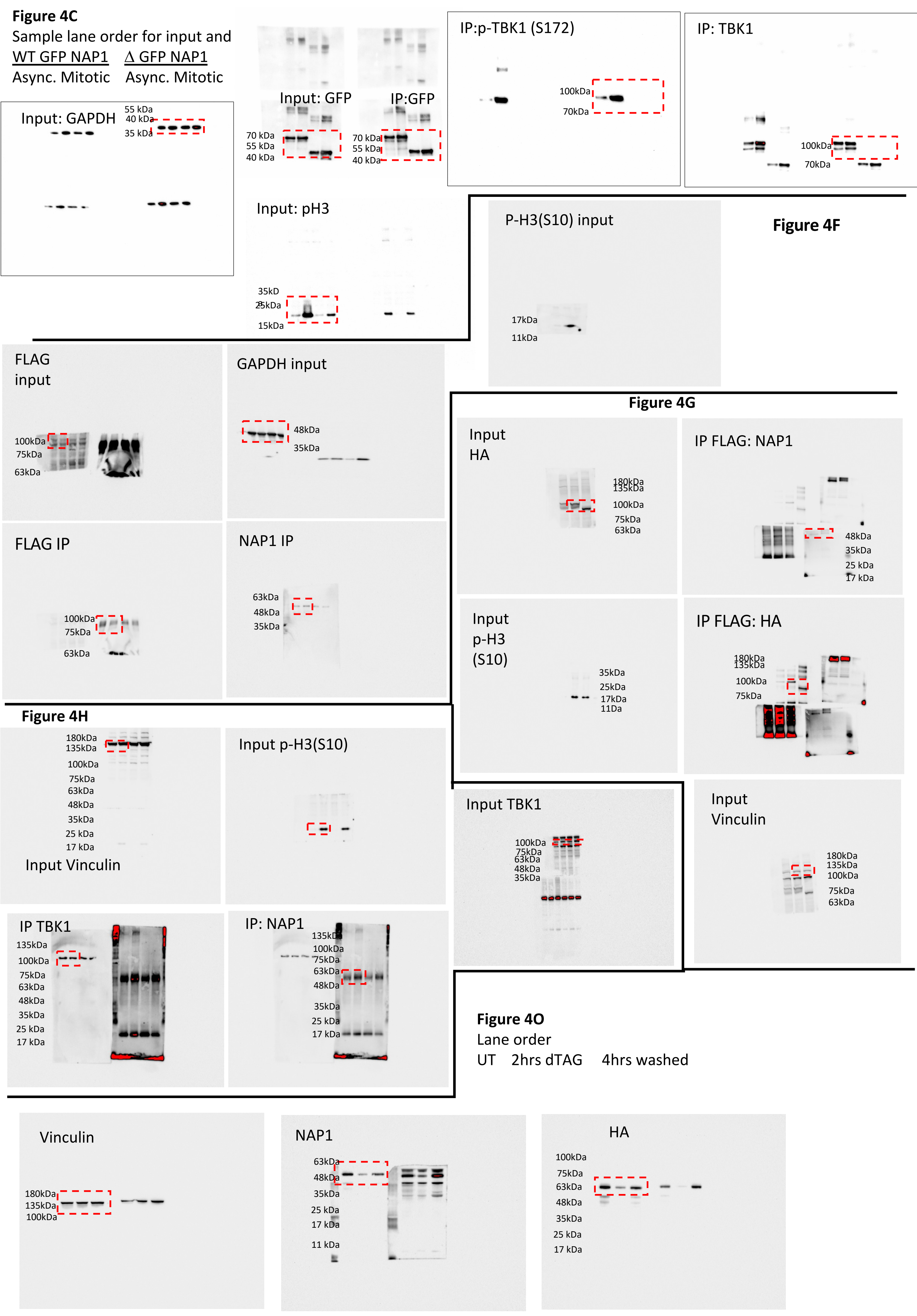
Figure 4C
Sample lane order for input and
WT GFP NAP1 Δ GFP NAP1
Async. Mitotic Async. Mitotic
Input: GAPDH
55 kDa
40 kDa
35 kDa
Input: GFP
70 kDa
55 kDa
40 kDa
IP:GFP
70 kDa
55 kDa
40 kDa
IP:p-TBK1 (S172)
100kDa
70kDa
IP: TBK1
100kDa
70kDa
Input: pH3
35kD
25kDa
15kDa
P-H3(S10) input
17kDa
11kDa
Figure 4F
FLAG
input
100kDa
75kDa
63kDa
GAPDH input
48kDa
35kDa
FLAG IP
100kDa
75kDa
63kDa
NAP1 IP
63kDa
48kDa
35kDa
Figure 4G
Input
HA
180kDa
135kDa
100kDa
75kDa
63kDa
IP FLAG: NAP1
48kDa
35kDa
25 kDa
17 kDa
Input
p-H3
(S10)
35kDa
25kDa
17kDa
11Da
IP FLAG: HA
180kDa
135kDa
100kDa
75kDa
Figure 4H
180kDa
135kDa
100kDa
75kDa
63kDa
48kDa
35kDa
25 kDa
17 kDa
Input Vinculin
Input p-H3(S10)
Input TBK1
100kDa
75kDa
63kDa
48kDa
35kDa
Input
Vinculin
180kDa
135kDa
100kDa
75kDa
63kDa
IP TBK1
135kDa
100kDa
75kDa
63kDa
48kDa
35kDa
25 kDa
17 kDa
IP: NAP1
135kDa
100kDa
75kDa
63kDa
48kDa
35kDa
25 kDa
17 kDa
Figure 4O
Lane order
UT 2hrs dTAG 4hrs washed
Vinculin
180kDa
135kDa
100kDa
NAP1
63kDa
48kDa
35kDa
25 kDa
17 kDa
11 kDa
HA
100kDa
75kDa
63kDa
48kDa
35kDa
25 kDa
17 kDa
