# Supplementary material for: NAK-associated protein 1/NAP1 activates TBK1 to ensure accurate mitosis and cytokinesis
Source: J Cell Biol. 2023 Dec 7;223(2):e202303082. doi: 10.1083/jcb.202303082 (PMC10702366; doi:10.1083/jcb.202303082)
Supplement: SourceData F5 — is the source file for Fig. 5. [file JCB_202303082_SourceDataF5.pdf]

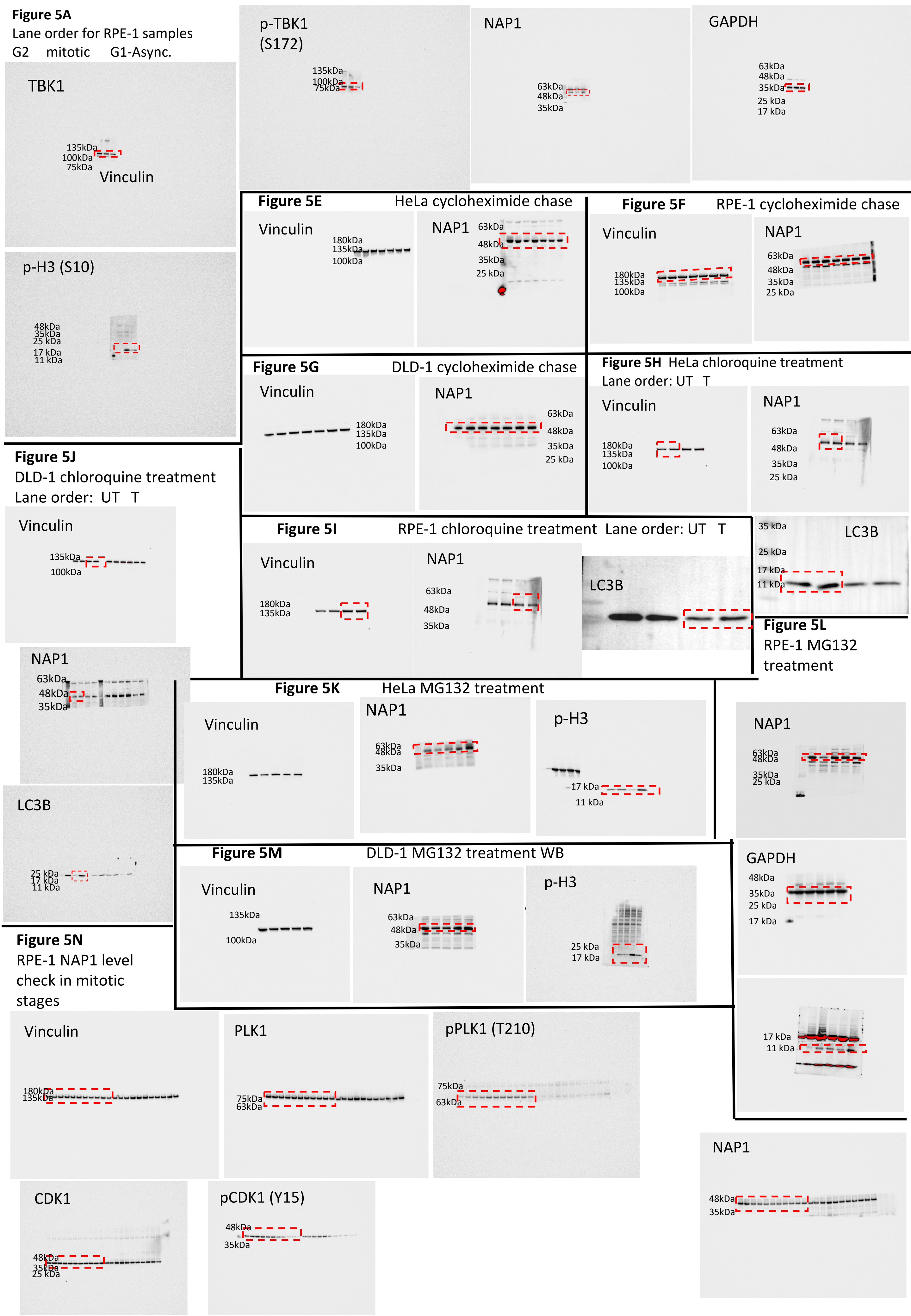

Figure 5A
Lane order for RPE-1 samples
G2 mitotic G1-Async.
TBK1
135kDa
100kDa
75kDa
Vinculin
p-TBK1 (S172)
135kDa
100kDa
75kDa
NAP1
63kDa
48kDa
35kDa
GAPDH
63kDa
48kDa
35kDa
25 kDa
17 kDa
p-H3 (S10)
48kDa
35kDa
25 kDa
17 kDa
11 kDa
Figure 5E
HeLa cycloheximide chase
Vinculin
180kDa
135kDa
100kDa
NAP1
63kDa
48kDa
35kDa
25 kDa
Figure 5F
RPE-1 cycloheximide chase
Vinculin
180kDa
135kDa
100kDa
NAP1
63kDa
48kDa
35kDa
25 kDa
Figure 5G
DLD-1 cycloheximide chase
Vinculin
180kDa
135kDa
100kDa
NAP1
63kDa
48kDa
35kDa
25 kDa
Figure 5H HeLa chloroquine treatment
Lane order: UT T
Vinculin
180kDa
135kDa
100kDa
NAP1
63kDa
48kDa
35kDa
25 kDa
Figure 5J
DLD-1 chloroquine treatment
Lane order: UT T
Vinculin
135kDa
100kDa
NAP1
63kDa
48kDa
35kDa
LC3B
25 kDa
17 kDa
11 kDa
Figure 5I
RPE-1 chloroquine treatment Lane order: UT T
Vinculin
180kDa
135kDa
NAP1
63kDa
48kDa
35kDa
LC3B
35 kDa
LC3B
25 kDa
17 kDa
11 kDa
Figure 5L
RPE-1 MG132 treatment
Figure 5K
HeLa MG132 treatment
Vinculin
180kDa
135kDa
NAP1
63kDa
48kDa
35kDa
p-H3
17 kDa
11 kDa
NAP1
63kDa
48kDa
35kDa
25 kDa
GAPDH
48kDa
35kDa
25 kDa
17 kDa
17 kDa
11 kDa
Figure 5M
DLD-1 MG132 treatment WB
Vinculin
135kDa
100kDa
NAP1
63kDa
48kDa
35kDa
p-H3
25 kDa
17 kDa
Figure 5N
RPE-1 NAP1 level check in mitotic stages
Vinculin
180kDa
135kDa
PLK1
75kDa
63kDa
pPLK1 (T210)
75kDa
63kDa
NAP1
48kDa
35kDa
CDK1
48kDa
35kDa
25 kDa
pCDK1 (Y15)
48kDa
35kDa
