# Supplementary figures and images for: NAK-associated protein 1/NAP1 activates TBK1 to ensure accurate mitosis and cytokinesis
Source: J Cell Biol. 2023 Dec 7;223(2):e202303082. doi: 10.1083/jcb.202303082 (PMC10702366; doi:10.1083/jcb.202303082)

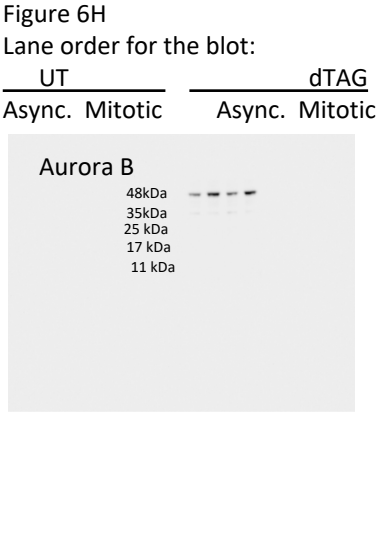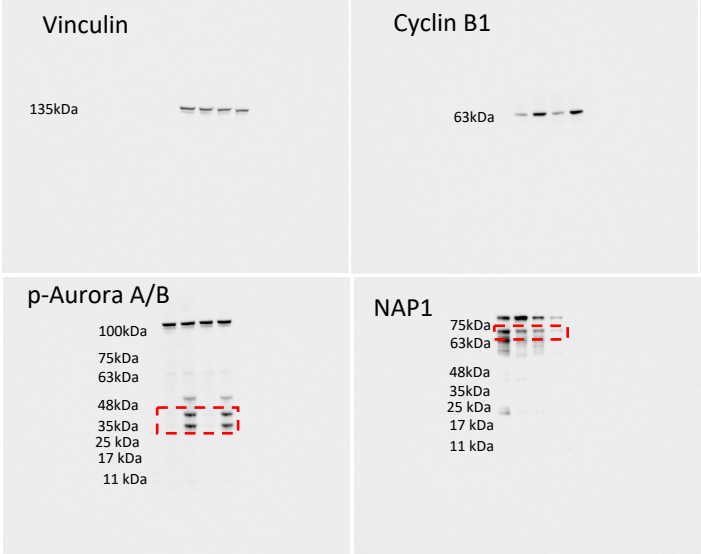

**Figure 6F**  
Lane order for the blot:

| WT HeLa |         | TBK1 KO |         |
|---------|---------|---------|---------|
| Async.  | Mitotic | Async.  | Mitotic |

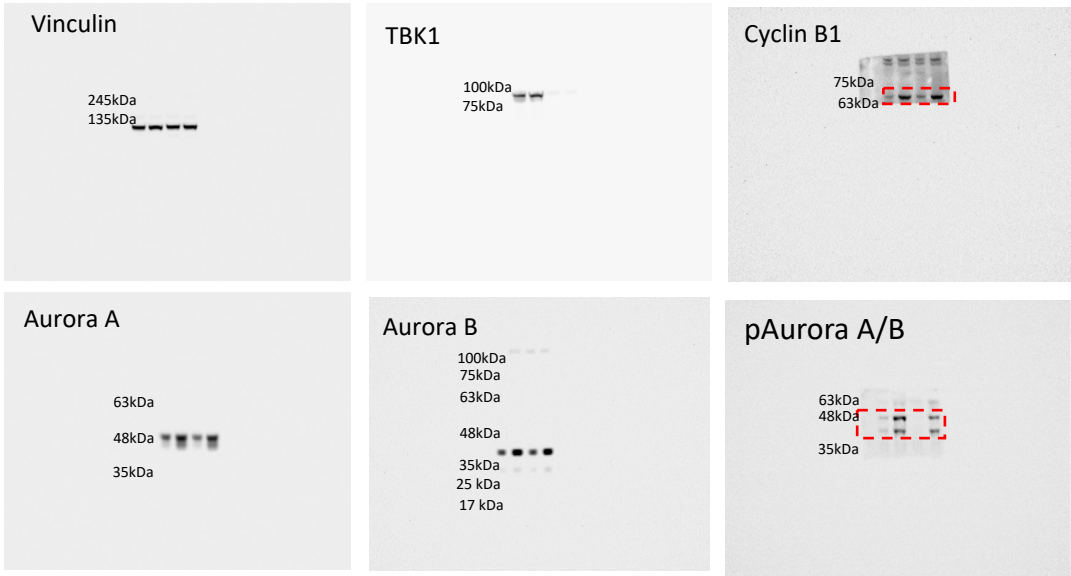

Supplement: SourceData F6 — is the source file for Fig. 6. [file JCB_202303082_SourceDataF6.pdf]

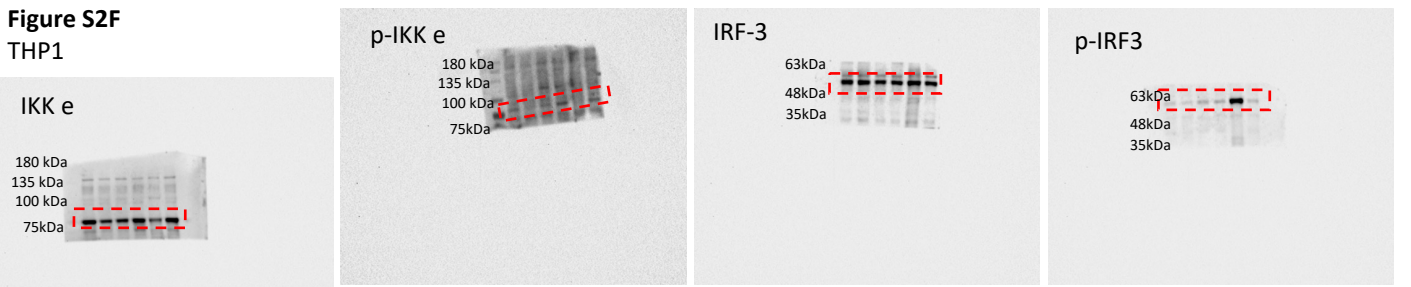

**Figure S2A**  
THP1 G1 G2 M

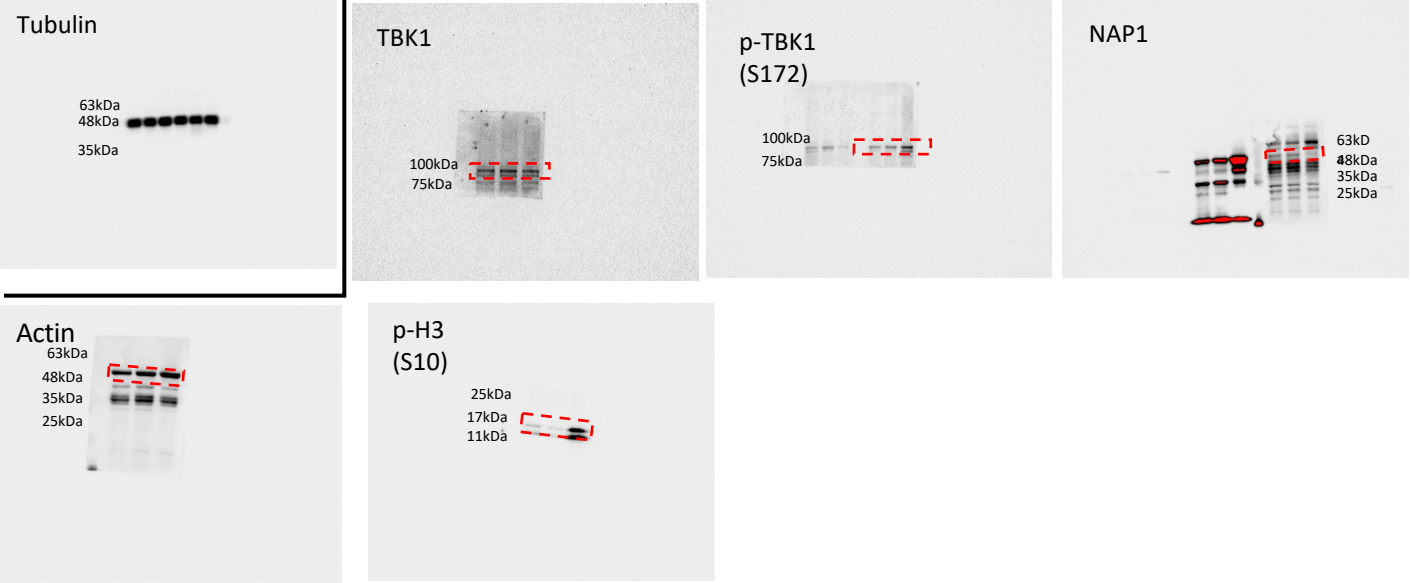

**Figure S2B**  
THP1 UT LPS PolyI:C

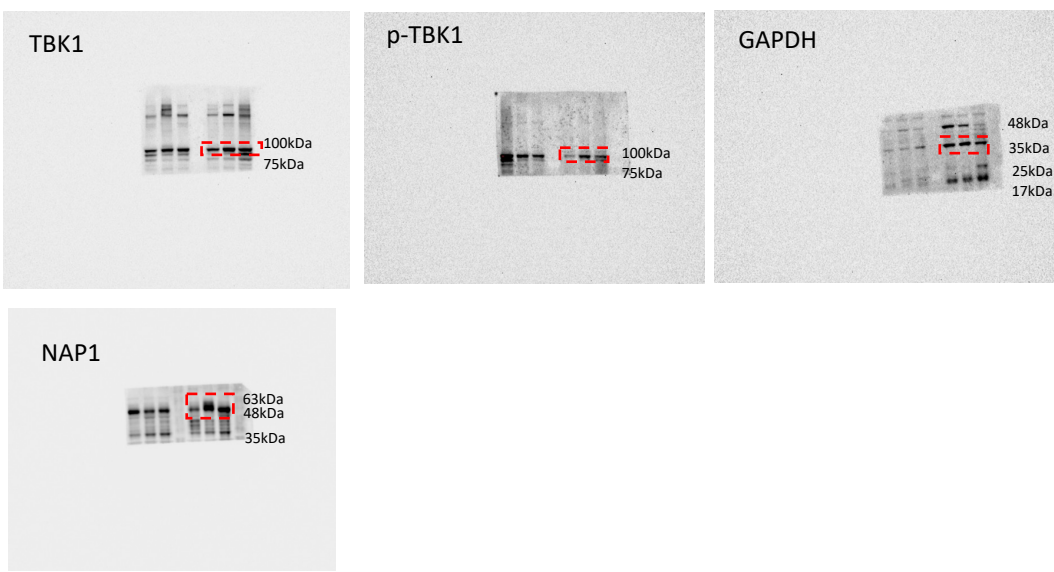

Supplement: SourceData FS2 — is the source file for Fig. S2. [file JCB_202303082_SourceDataFS2.pdf]
