# Supplementary material for: NAK-associated protein 1/NAP1 activates TBK1 to ensure accurate mitosis and cytokinesis
Source: J Cell Biol. 2023 Dec 7;223(2):e202303082. doi: 10.1083/jcb.202303082 (PMC10702366; doi:10.1083/jcb.202303082)
Supplement: SourceData FS1 — is the source file for Fig. S1. [file JCB_202303082_SourceDataFS1.pdf]

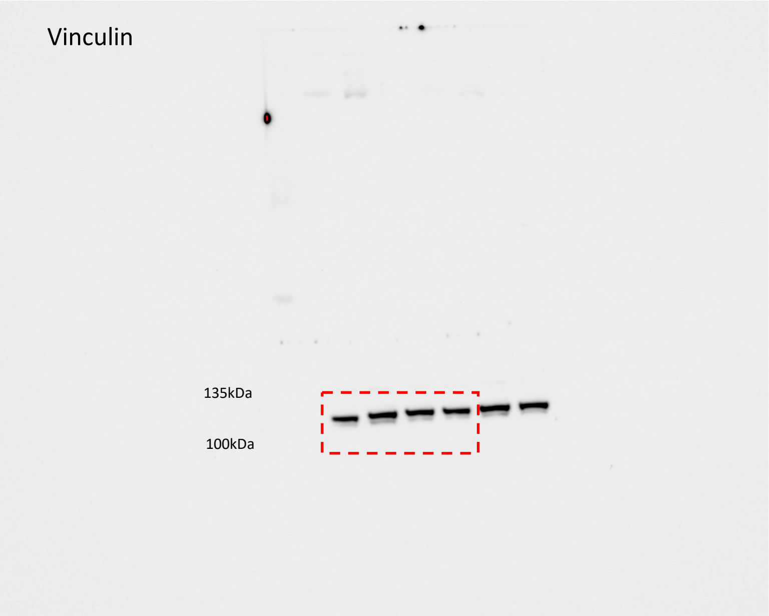

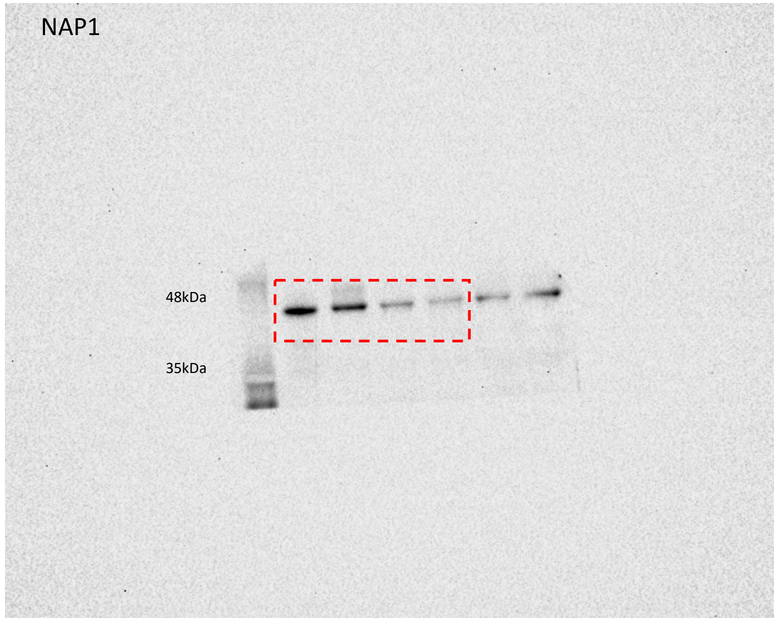

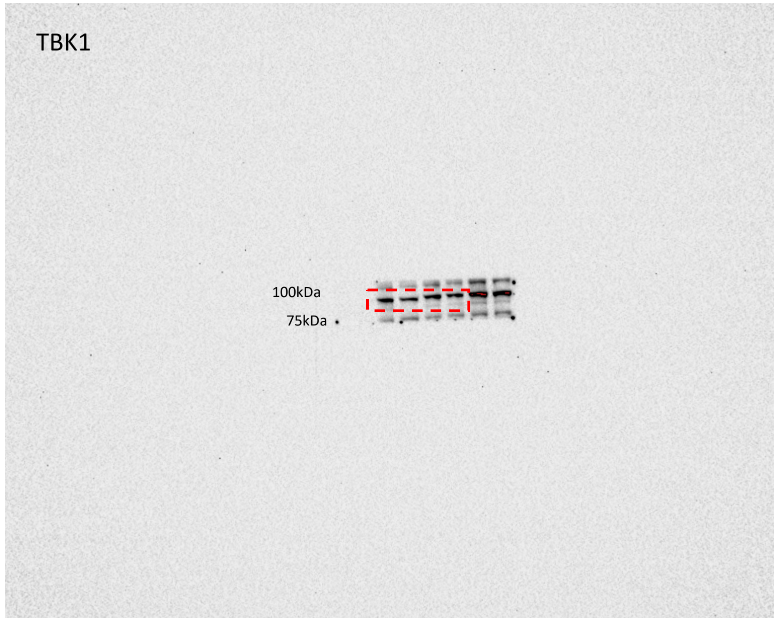

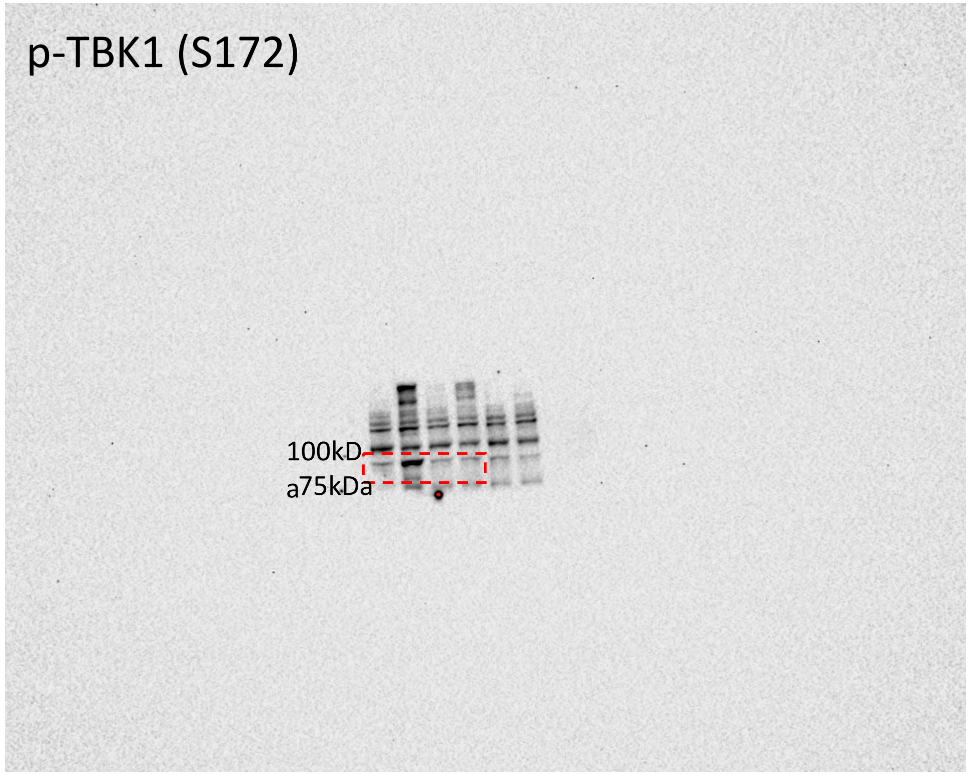

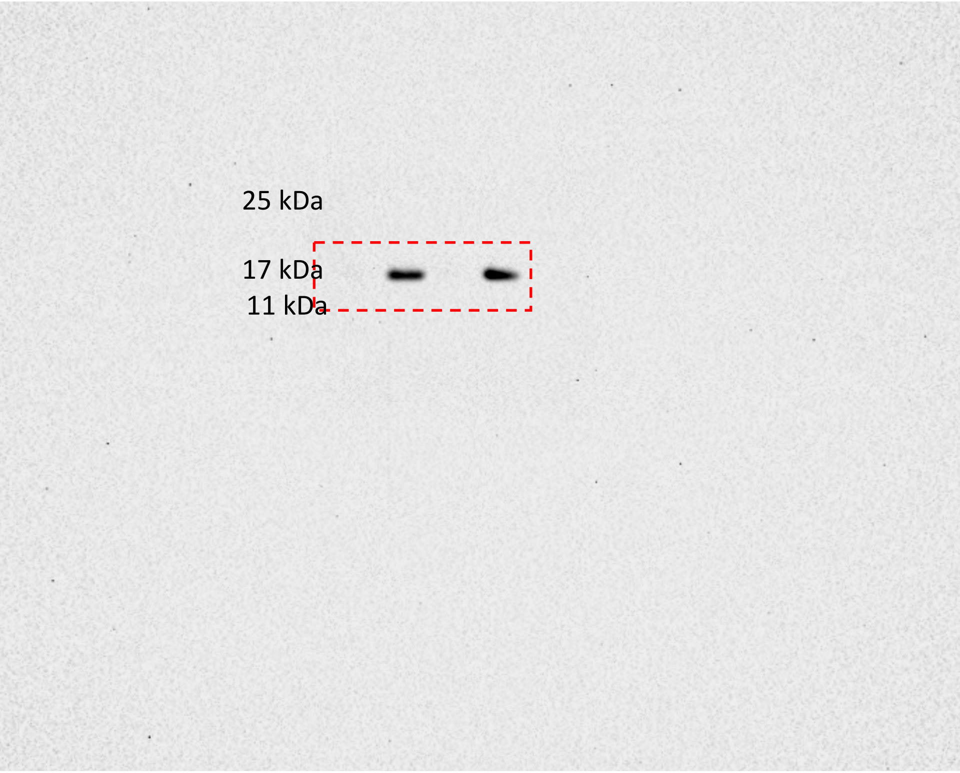

NAP1 KD DLD-1

Lane order for the blot:

<u>Scramble ShRNA</u> <u>NAP1 KD ShRNA</u>

Async. Mitotic Async. Mitotic

Figure S1
